# Supplementary material for: All-optical nonreciprocity due to valley polarization pumping in transition metal dichalcogenides
Source: Nat Commun. 2021 Jun 18;12:3746. doi: 10.1038/s41467-021-24138-0 (PMC8213841; doi:10.1038/s41467-021-24138-0)
Supplement: Supplementary file 1 — Supplementary information [file 41467_2021_24138_MOESM1_ESM.docx]

**Supplementary information**

**All-optical nonreciprocity due to valley polarization pumping in transition metal dichalcogenides**

Sriram Guddala^1^, Yuma Kawaguchi^1^, Filipp Komissarenko^1^, Svetlana Kiriushechkina^1^, Anton Vakulenko^1^, Kai Chen^1,2^, Andrea Alù^3,2,1^, Vinod Menon^4,2^, and Alexander B. Khanikaev^1,2,4^^✉^

^1^Department of Electrical Engineering, Grove School of Engineering, City College of the City University of New York, 140th Street and Convent Avenue, New York, NY 10031, USA.

^2^Physics Program, Graduate Center of the City University of New York, New York, NY 10016, USA.

^3^Photonics Initiative, Advanced Science Research Center, City University of New York, New York, NY 10031, USA

^4^Department of Physics, City College of New York, 160 Convent Ave., New York, NY 10031, USA

^✉^email: [akhanikaev@ccny.cuny.edu](mailto:akhanikaev@ccny.cuny.edu)

**Supplementary Note 1**

**1.1 Analytical models to fit the experimental results**

The reflectivity spectra for $\sigma^{-}$ and $\sigma^{+}$probes at maximum pump power (Fig. 2**d** of the Main Text) was fitted by the Fresnel equation modified by sheet conductivity^1^,

$\left| r \right|^{2}=\left| \frac{n_{2}-n_{1}-Z_{0}\tilde{\sigma}}{n_{2}+n_{1}-Z_{0}\tilde{\sigma}} \right|^{2}$. (1.1)

For $\sigma^{-}$ and $\sigma^{+}$probes reflectivities, one considers the sheet conductivities $\tilde{\sigma}_{CW}=\sigma_{xx}+\sigma_{xy}$,$\tilde{\sigma}_{CCW}=\sigma_{xx}-\sigma_{xy}$, respectively, which yield

$\left| r_{CW} \right|^{2}=\left| \frac{n_{2}-n_{1}-Z_{0}(\sigma_{xx}+\sigma_{xy})}{n_{2}+n_{1}-Z_{0}(\sigma_{xx}+\sigma_{xy})} \right|^{2}$, (1.2)

$\left| r_{CCW} \right|^{2}=\left| \frac{n_{2}-n_{1}-Z_{0}(\sigma_{xx}-\sigma_{xy})}{n_{2}+n_{1}-Z_{0}(\sigma_{xx}-\sigma_{xy})} \right|^{2}$, (1.3)

where $Z_{0}=\sqrt{{\mu_{0}}/{\epsilon_{0}}}$ is the free space impedance, and $n_{1}$, $n_{2}$ are the refractive indices of the superstrate and substrate surrounding the TMDs monolayer, respectively. The off-diagonal component $\sigma_{xy}$ gives rise to the difference in reflectivities of the $\sigma^{-}$ and $\sigma^{+}$probes.

The optical permittivity and sheet conductivity of TMD monolayer, which are the functions of the photon energy $E$, were fitted by the Lorentzian dispersion model^2^,

$\epsilon\left( E \right)=1+\frac{f}{E_{ex}^{2}-E^{2}-iE\gamma}$, (1.4)

$\tilde{\sigma}\left( E \right)=-\frac{i\epsilon_{0}Ed}{h}\left[ \epsilon\left( E \right)-1 \right]=-\frac{i\epsilon_{0}Ed}{h}\frac{f}{E_{ex}^{2}-E^{2}-iE\gamma}$, (1.5)

where $f$ is the oscillator strength, $E_{ex}$ is the exciton resonance energy and $\gamma$ is the linewidth of the exciton resonance, and in general are the pump intensity dependent parameters, while $d$ is the thickness of the TMD monolayer and $h$ is the Planck constant. As an example, the respective parameters for the case of pump power $P=450 \mu W$ are given in Supplementary Table 1.1.

**Supplementary Table 1.1.** Fitting parameters of Lorentzian type model in Fig.**2** for $\sigma^{-}$ pump

|  | $f (eV^{2})$ | $E_{ex} (\mathrm{eV})$ | $\gamma(\mathrm{eV})$ | $d (\mathrm{nm})$ |
| --- | --- | --- | --- | --- |
| ${+k}_{z} prob (\sigma^{+})$ | 61.59 | 2.0155 | 0.040 | 0.618 |
| ${-k}_{z} prob (\sigma^{+})$ | 70.95 | 2.0135 | 0.040 |  |
| ${+k}_{z} prob (\sigma^{-})$ | 73.13 | 2.0115 | 0.040 |  |
| ${-k}_{z} prob (\sigma^{-})$ | 55.03 | 2.0140 | 0.041 |  |

**1.2 Perturbation theory for the waveguide interacting with the dichroic 2D material**

The simulation of the waveguide was performed in COMSOL Multiphysics. Experimentally obtained optical conductivities were then used to obtain the imaginary parts of the propagation constant in the waveguide $\beta$, which describes the attenuation of the guided wave, and is given by the expression:

$\mathrm{Im}\left( \beta\right)=\frac{\beta_{0}}{{\omega W}_{0}}\int_{TMD} {dS[\mathbf{E}}_{0}^{*}{Re(\hat{\sigma}}_{TMD})\mathbf{E}_{0}]$, (1.6)

where

$W_{0}=2\int_{V} dV[\left| E_{0}\left( r \right) \right|^{2}\epsilon_{0}\epsilon_{r}(r)]$, (1.7)

and ${Re(\hat{\sigma}}_{TMD})$ is the real part of the optical conductivity of the TMDs monolayer, $\mathbf{E}_{0}$ is the absolute value of the electric field, and $W_{0}$ is the energy density of the guided wave (the core plus the cladding). The imaginary part of the propagation constant $\alpha$ due to the diagonal component of conductivity tensor, i.e. non-discriminating handedness of the field helicity, is then given by

$\alpha=\frac{\beta_{0}}{{\omega W}_{0}}\int_{TMD} dS[{\left| E_{0} \right|^{2}\sigma}_{xx}]$, (1.8)

while the helicity dependent absorption differential is defined by the off-diagonal component of conductivity as

$\delta=\frac{\beta_{0}}{{2\omega W}_{0}}\int_{TMD} dS[{\left| E_{0CW} \right|^{2}-\left| E_{0CW} \right|^{2})(\sigma}_{K}-\sigma_{K^{'}})]$. (1.9)

Since the conductivity is uniform across the TMD monolayer, the respective terms can be moved out from the integrals and will appear as factors. Then, the integrals defining the nonreciprocal absorption are evaluated from numerically calculated field profiles with values given in Supplementary Table 1.2.

**Supplementary Table 1.2**| Values of the integrals for the perturbation theory used for evaluation of the propagation constant

| Expression | Evaluated integration value |
| --- | --- |
| $\int_{TMD} dS\left\vert E_{0CW} \right\vert^{2}$ | 189.1 V^2^ |
| $\int_{TMD} dS\left\vert E_{0CCW} \right\vert^{2}$ | 1029.3 V^2^ |
| $\int_{core} dV\left\vert E_{0}(r) \right\vert^{2}$ | 0.000419 V^2^m |
| $\int_{clad} dV\left\vert E_{0}(r) \right\vert^{2}$ | 0.00111 V^2^m |


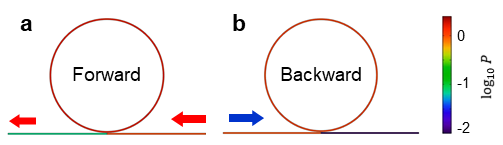


Supplementary Figure 1. **Power profiles of the waveguide and ring resonator**. **a** and **b**, power profiles based on couple mode theory modeling for forward and backward, respectively.

**Supplementary Table 1.3**. Normalized power in resonator and output waveguide

|  | $P_{ring}/P_{in}$ | $P_{out}{/P}_{in}$ |
| --- | --- | --- |
| Forward | 1.4760 | 0.0694 |
| Backward | 1.0415 | 0.000644 |

**Supplementary Note 2**

**2.1 Schematic of the sample**

# The WS_2_ monolayer encapsulated between two thin hBN layers of $7 \mathrm{nm}$ each. The hBNs and WS_2_ monolayers were transferred to 120 $\mu m$ thick BK7 glass substrate one on top of the another. The environment of the sample made symmetric further by coating with a thick PMMA A11 (MicroChem, $n_{\mathrm{PMMA}}=1.5$) of$1.7 \mu m$, which has same refractive index as the bottom glass substrate ($n_{BK7}= 1.5$).


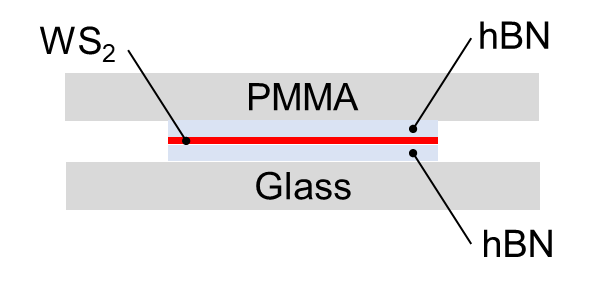


Supplementary Figure 2. **Schematic of the sample.** WS_2_ monolayer is encapsulated between two thin hBN layers and symmetrically sandwiched between materials (BK7 glass cover slip and thick PMMA slab) of the same refractive index. Red: WS_2_ monolayer; light blue: hBN layers.

**2.2 Transfer matrix method fitting of reflectance measurements**

The pump power dependent surface conductivity of the encapsulated WS_2_ monolayer were obtained by fitting the reflectance spectra for different pump powers. The reflectance curves of encapsulated WS_2_ monolayer show Fabry-Perot modes due to the finite thickness of the PMMA film shown in Supplementary Fig. 3. We implemented transfer matrix method (TMM) with the inclusion of boundary conditions based on Eq. (1.1) and Eq. (1.5) to fit the spectral reflectance of the encapsulated WS_2_ monolayer to extract its surface conductivity and account for Fabry-Perot resonances. The reflectance from the region without WS2 and with double hBN layer was used to extract the respective hBN contribution to the conductivity and was also used in our fitting of the surface conductivity of WS_2_ monolayer by the transfer matrix method.

Examples of reflectance spectra for both hBN stack: glass/hBN/hBN/PMMA and WS_2_ monolayer region: glass/hBN/WS_2_/hBN/PMMA at zero pump power are shown below in Supplementary Fig. 3. The fitting parameters based on Eq. (1.5) for the sheet conductivity at zero pump power were found to be $E_{\mathrm{ex}}=2.017 \mathrm{eV}$, $f=8.6 eV^{2}$ and $\gamma=0.021 \mathrm{eV}$. The extracted wavelength dependent sheet conductivity, real and imaginary parts, are also shown in Supplementary Fig. 3. The same procedure was used for pump power dependent reflectance data and extracted wavelength dependent real and imaginary parts of surface conductivity components (Fig. 3 of the Main Text) for increasing pump power.


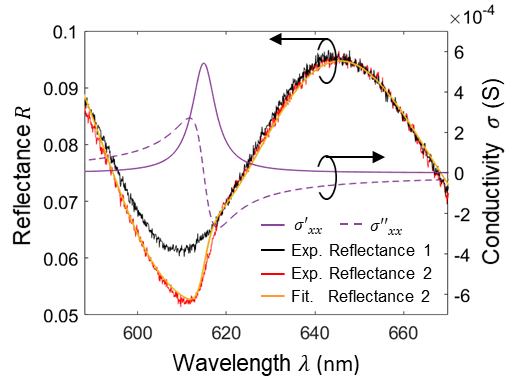


Supplementary Figure 3. **Reflectance and sheet conductivity. Black line:** Reflectance spectra of hBN stack region: PMMA/hBN/hBN/SiO_2_. **Red line**: encapsulated WS_2_ monolayer region: PMMA/hBN/WS_2_/hBN/SiO_2._. **Orange line**: TMM fitted curve for encapsulated WS_2_ region. Extracted real (solid) and imaginary (dashed) diagonal component of sheet conductivity are shown by purple colour.

**2.3 Photoinduced nonreciprocal reflectivity response of WS_2_ monolayer**

The photoinduced nonreciprocal reflectance from the sample with WS_2_ monolayer was investigated by measuring the reflectance for circularly polarized probe beams with two opposite wave vectors (corresponding to forward and backward propagation) for the same handedness of the pump. Each probe beam direction of propagation and helicity dependent reflectivity data for increasing pump power are given below.

The power dependent reflectance for $\sigma^{-}$ helicity probe (and $\sigma^{+}$ helicity pump) for both forward and backward directions are shown in Supplementary Figs. 4, **a** and **c**, respectively. Similarly, Supplementary Figs. 4, **b** and **d**, show the power dependent reflectance for $\sigma^{-}$ helicity probe (and $\sigma^{-}$ helicity pump) for both forward and backward directions, respectively. The difference in spectral reflectance intensity for changed probe beam direction under same helicity pump indicates the nonreciprocal character pf reflectance $R\left( +k_{z} \right)\neq R(-k_{z})$. It can be noticed that the response is reversible between the propagation directions by changing the helicity of the pump beam. The reflectance $R/R_{0}$ was obtained by normalizing the pump power dependent reflection intensity to the zero-pump power reflection intensity.


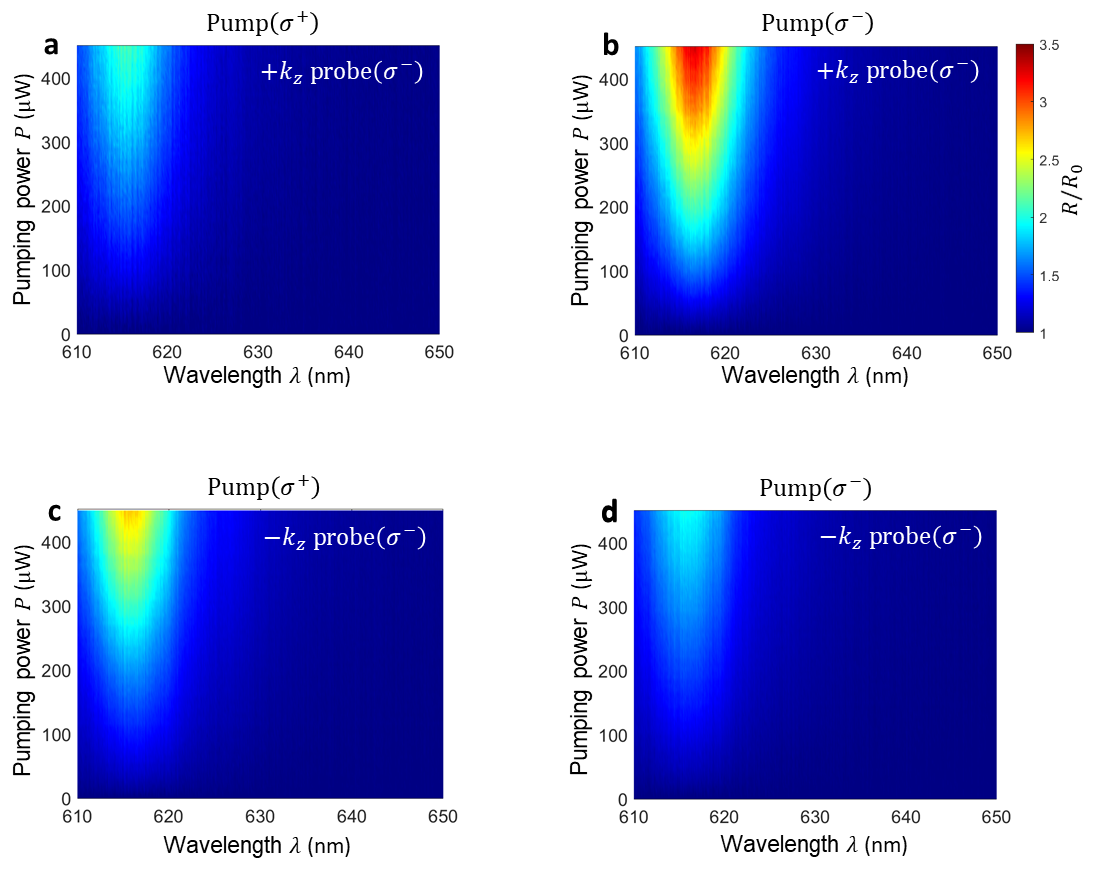


# Supplementary Figure 4. **Pump power dependent reflectance (**$\boldsymbol{R/}\boldsymbol{R}_{\boldsymbol{0}}\boldsymbol{)}$**of** $\boldsymbol{\sigma}^{\boldsymbol{-}}$ **probe. a**, and **c**, $\sigma^{-}$ probe beam reflectivity for forward $\left( +k_{z} \right)$ and backward $\left( -k_{z} \right)$ propagation directions, respectively, for increasing $\sigma^{+}$ pump power. **b**, and **d**,$\sigma^{-}$ probe beam reflectivity for forward $\left( +k_{z} \right)$ and backward $\left( -k_{z} \right)$ propagation directions, respectively, for increasing $\sigma^{-}$ pump power.

# Similarly, Supplementary Figs. 5, **a** and **c**, show the power dependent reflectance for $\sigma^{+}$ helicity probe beam for pump of $\sigma^{+}$ helicity for both forward and backward directions, respectively. Finally, Supplementary Figs. 5 **b** and **d** show the power dependent reflectance for $\sigma^{+}$ helicity probe for pump of $\sigma^{-}$ helicity for both forward and backward directions, respectively.


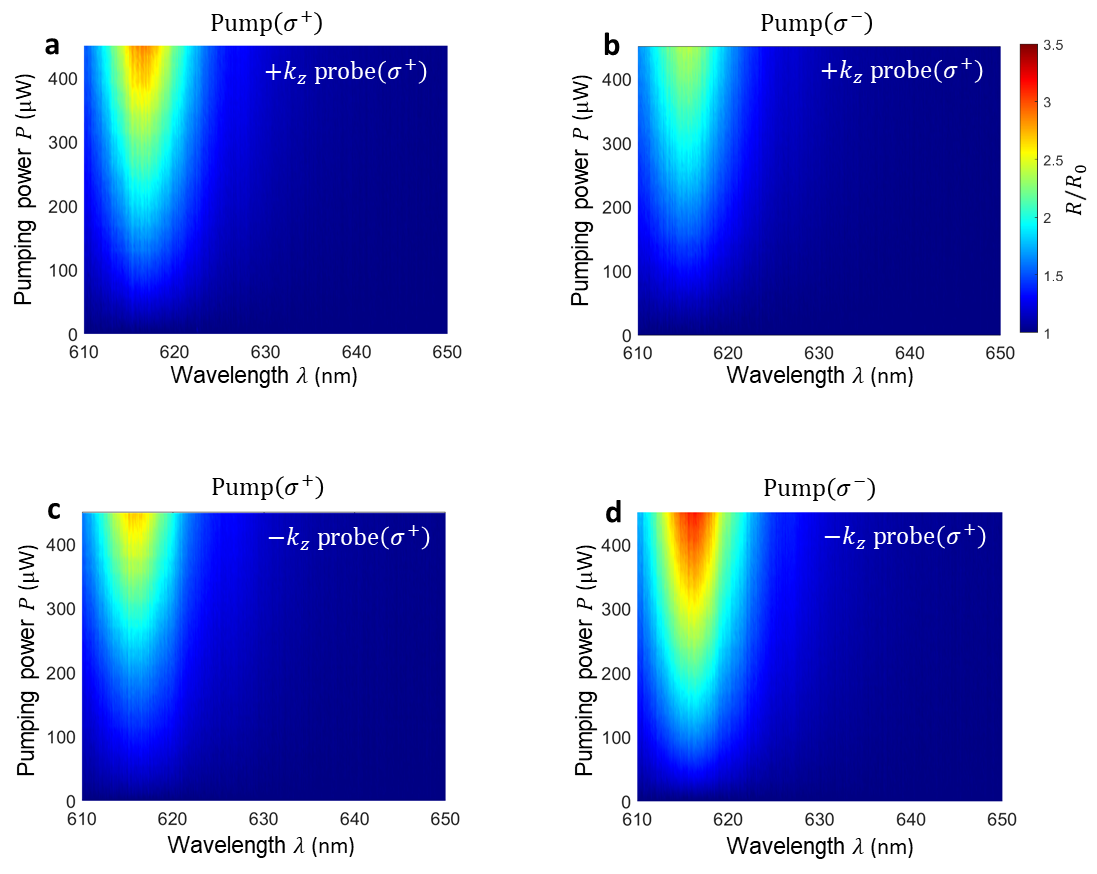


# Supplementary Figure 5. **Pump power dependent reflectance (**$\boldsymbol{R/}\boldsymbol{R}_{\boldsymbol{0}}\boldsymbol{)}$**of** $\boldsymbol{\sigma}^{\boldsymbol{+}}$ **probe. a**, and **c**, $\sigma^{+}$ probe beam reflectivity for forward $\left( +k_{z} \right)$ and backward $\left( -k_{z} \right)$ propagation directions, respectively, for increasing $\sigma^{+}$ pump power. **b**, and **d**,$\sigma^{+}$ probe beam reflectivity for forward $\left( +k_{z} \right)$ and backward $\left( -k_{z} \right)$ propagation directions, respectively, for increasing $\sigma^{-}$ pump power.

**2.4 Photoinduced nonreciprocal circular dichroism response of WS_2_ monolayer with** $\boldsymbol{\sigma}^{\boldsymbol{+}}$ **pump**

The degree of photoinduced nonreciprocal circular dichroism is calculated, by using Eq. 4 in the Main Text, from the reflectivity data for two opposite helicity probe beams propagating in the same direction. The increase in pump power results in increase of the degree of circular dichroism due to increased population of valley polarized excitons at specific valley, i.e., the valley polarization of excitons as can be seen in Supplementary Fig. 6.


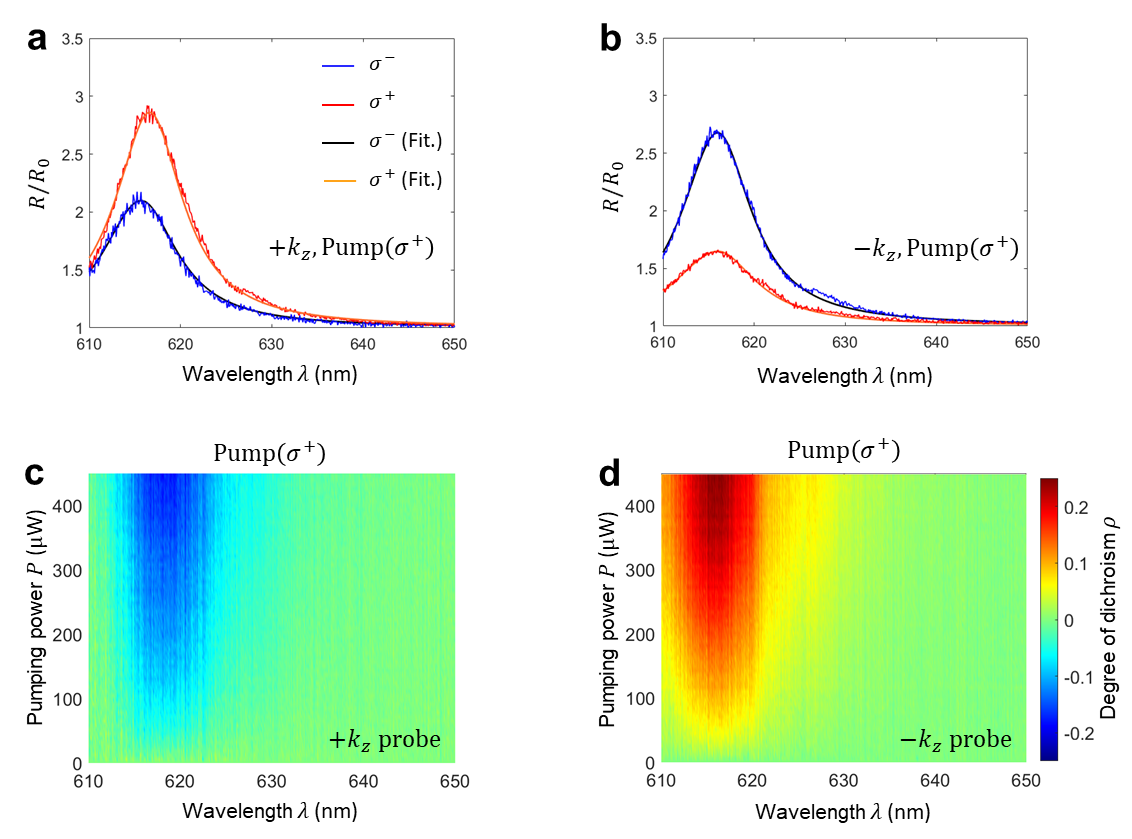


# Supplementary Figure 6. **Experimental demonstration of photoinduced nonreciprocal circular dichroism in WS_2_ for the** $\boldsymbol{\sigma}^{\boldsymbol{+}}$ **pump**. **a** and **b**, show cases of ($\sigma^{+}$ and $\sigma^{-}$) probes spectral reflectivity for $k=k_{z}$ and $k=-k_{z}$ cases, respectively. Solid lines show the results of fitting by a modified Fresnel equation with surface conductivity described by a Lorentzian model in Supplement A. **c** and **d**, show pump power dependent dichroic response as seen in reflectivity from the sample for probes coming from opposite directions.

**Table 2.1**| Fitting parameters of Lorentzian type model in Supplementary Figs. 6**a,b** for $\sigma^{+}$ pump

|  | $f (eV^{2})$ | $E_{ex} (\mathrm{eV})$ | $\gamma(\mathrm{eV})$ | $d (\mathrm{nm})$ |
| --- | --- | --- | --- | --- |
| ${+k}_{z} prob (\sigma^{+})$ | 68.06 | 2.0120 | 0.040 | 0.618 |
| ${-k}_{z} prob (\sigma^{+})$ | 56.69 | 2.0150 | 0.040 |  |
| ${+k}_{z} prob (\sigma^{-})$ | 47.70 | 2.0145 | 0.041 |  |
| ${-k}_{z} prob (\sigma^{-})$ | 65.88 | 2.0140 | 0.040 |  |

**2.5 Nonreciprocal transmittance spectra of** $\boldsymbol{\sigma}^{\boldsymbol{-}}$ **probe**

Nonreciprocal circular dichroism of WS_2_ monolayer was also measured in transmission mode using the same experimental set up shown in Fig. 1**e** of the Main Text. The schematic of the transmission measurement geometry is shown below in Supplementary Fig. 7. The $\sigma^{-}$ helicity probe beam transmission in both forward ( ${+k}_{x}$) and backward (${-k}_{x}$) directions show difference in transmission for increasing $\sigma^{-}$ helicity pump power as shown in Supplementary Fig. 7. The spectral transmittance of probe beams in both forward and backward directions at maximum pump power of 450 $\mu$W is shown in Supplementary Fig. 7**b**. It can be noticed that the transmittance at the exciton resonance position decreases for forward direction probe in comparison to the backward direction probe even though both beams are of the same $\sigma^{-}$ helicity. It is a clear indication of nonreciprocity response in monolayer WS_2_ due to nonreciprocal circular dichroism and proves the ability of even a single monolayer to perform partial optical isolation under optical bias without any external magnetic field.


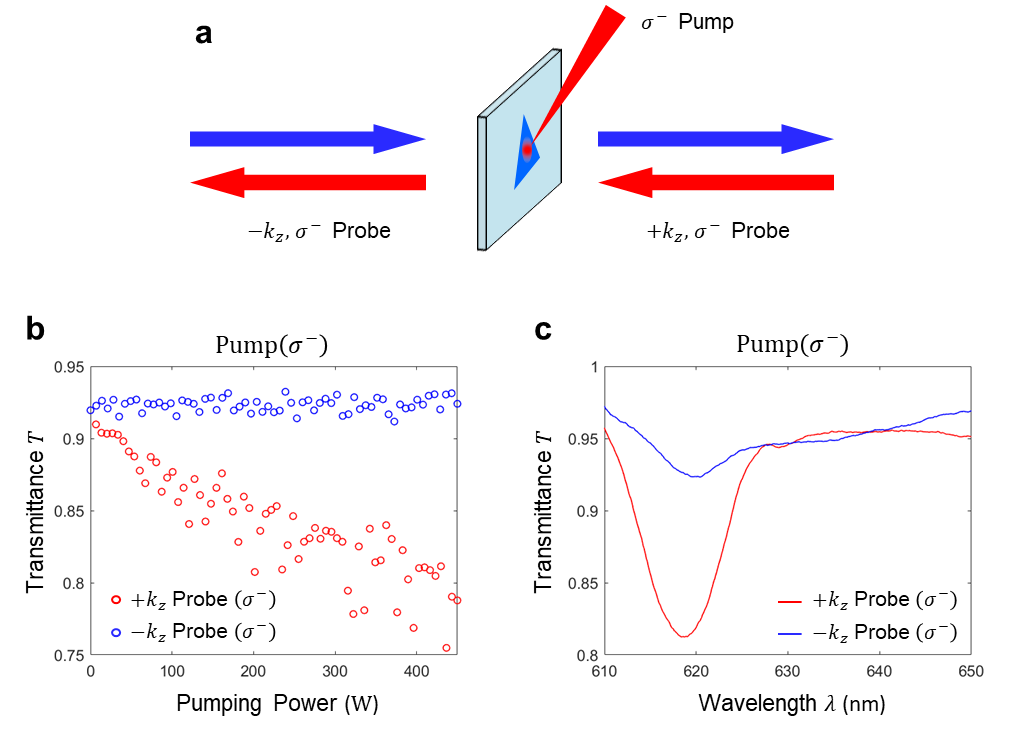


Supplementary Figure 7. **Experimental demonstration of photoinduced nonreciprocal transmission in WS_2_**.**a,** Schematic of the nonreciprocal transmission measurement scheme. **b**, Pump ($\sigma^{-}$ helicity) power dependent optical transmission at 617 nm for $\sigma^{-}$ probe beams in both forward and backward directions. **c**, Wavelength dependence of nonreciprocal transmission of $\sigma^{-}$ probe beam at maximum pump power (450 $\mu$W).

**2.6 Linear polarization reflection**

To confirm that the dichroism is indeed induced by the circularly polarized pump, we also performed measurement for linearly polarized pump in reflection geometry with the wave vector inversion. As expected, no dichroism was observed. The respective results are given in Supplementary Fig. 8.


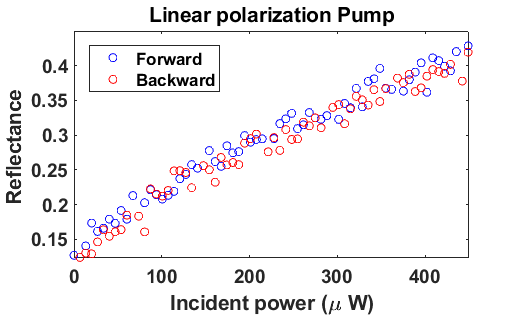


Supplementary Figure 8. **Absence of the dichroic response under the linearly polarized pump.**

**2.7 Integrated all-optical isolator with switchable isolation direction**

Unlike the magnets which are bulky, the pumping laser can be fully integrated on chip, which paves the way toward fully integrated nonreciprocal photonic systems. Indeed, the effect observed appears at rather moderate pump fluences. In our experiment we have 450 µW maximal power pump beam focused on 25 µm x 25 µm area, which, for the integrated setup in Fig. 4 of the Main Text with TMD of size 3.3 µm by 50 nm implies the reduction of power by ~3,000 times, which corresponds to the power of only 0.2 µW. This power can be farther reduced by using an integrated scheme in Supplementary Fig. 9, where we propose to use the pump field enhancement in the ring resonator. Such fully integrated as in Supplementary Fig. 9 setup has clear advantages over current magnetic nonreciprocal devices. More importantly, it also offers tunability by switching on/off the pumps, effectively yielding an all-optical control of the nonreciprocal device operation.


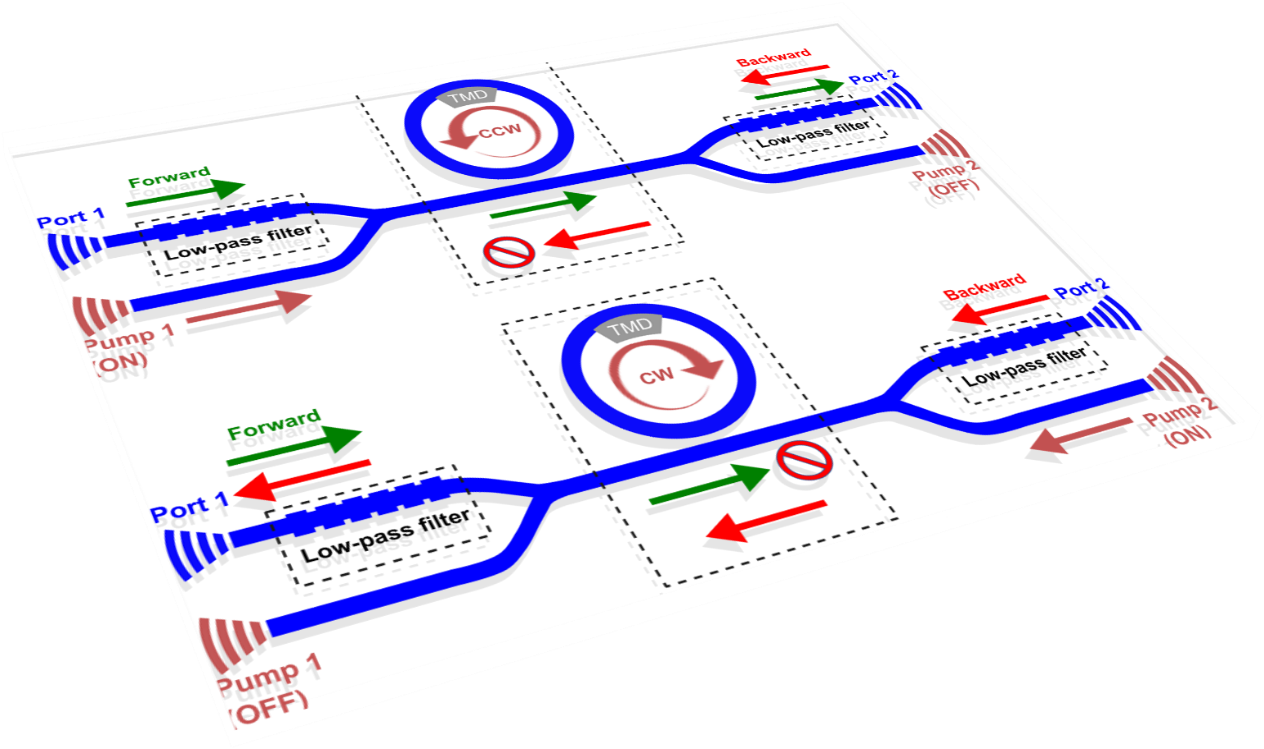


Supplementary Figure 9. **Schematic of all-optical isolator with switchable isolation direction**. Top and bottom schemes show cases of backward and forward isolation, respectively. Control of directionality is achieved by pumping at ports Pump 1 or Pump 2, which leads to the modes with opposite propagation direction and chiral near-field of opposite handedness in the ring resonator. No pump would yield reciprocal (bidirectional) regime.

**2.8 Suggested mechanism of the photoinduced circular dichroism**

The fact that the dichroism does not vanish over long measurement times (compared to the pump duration) indicates the presence of long-living valley-polarized excitations in the system. Indeed, as mentioned in the Main Text, while the excitons in TMDs are known to have rather short lifetimes of less than 2 ps, recent time-resolved pump-probe experimental studies suggest that the lifetime of photoexcited free carriers can very long. We therefore attribute the large value of the measured dichroism to the delayed relaxation of the photoexcited valley-polarized free carriers into exciton states with partial preservation of the valley-polarization. In the proposed scenario, the valley-preserving relaxation of free-carriers leads to a larger density of valley-polarized excitons for strong intensities of CP pump. This mechanism is evidenced by an onset of saturation at the respective valley due to the exciton-exciton interactions, which is clearly seen in Fig. 3 of the Main Text. At the same time, the saturation due to exciton-exciton interactions at the opposite (unpumped) valley was found to have a relatively late onset, which further confirms the role of photoinduced valley polarization as the main mechanism of the observed circular dichroism. As an illustration of the proposed mechanism, Supplementary Fig. 10 shows a schematic microscopic picture of the suggested processes**.**


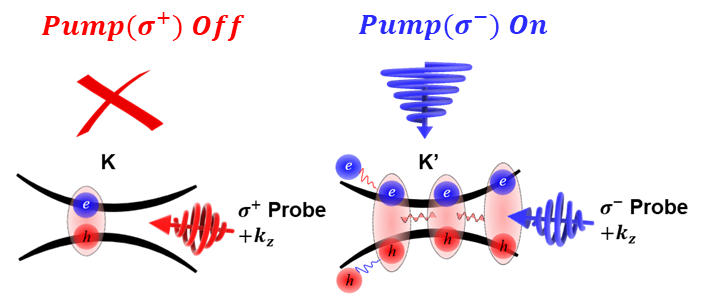


Supplementary Figure 10. **Suggested mechanism of giant photoinduced circular dichroism.** Long-living free carriers with preserved valley polarization relax into short-lived exciton states responsible for the enhanced absorption of incident probe signal of a particular handedness, and thus giving rise to the dichroic response.

**Supplementary References**

1. Stauber, T., Peres, N. M. R. & Geim, A. K. Optical conductivity of graphene in the visible region of the spectrum. *Phys. Rev. B - Condens. Matter Mater. Phys.* **78**, 085432 (2008).

2. Li, Y. *et al.* Measurement of the optical dielectric function of monolayer transition-metal dichalcogenides : *Phys. Rev. B* **205422**, 1–6 (2014).
